# Hyaluronic acid filler for lip augmentation in patients with systemic sclerosis: A prospective cohort study

**DOI:** 10.1016/j.jdcr.2024.05.006

**Published:** 2024-05-16

**Authors:** Madisen Swallow, May Elgash, Sa Rang Kim, Monique Hinchcliff, Kathleen Suozzi

**Affiliations:** a Yale School of Medicine, New Haven, Connecticut; b Department of Dermatology, Yale School of Medicine, New Haven, Connecticut; c Department of Rheumatology, Yale School of Medicine, New Haven, Connecticut

**Keywords:** clinical research, connective tissue disease, hyaluronic acid filler, systemic sclerosis

Orofacial symptoms are common in patients with systemic sclerosis (SSc) and thin lips are a common cutaneous concern.^1^ Hyaluronic acid (HA) fillers are used for lip volume restoration but data on their efficacy and safety in patients with SSc are limited.^2^^,^^3^ In patients with connective tissue disorders, treatment of cosmetic concerns with HA fillers have long been avoided due to concern for disease exacerbation. However, in patients with lupus, none of the studies report disease reactivation after treatment with HA fillers.^2^ HA fillers with high degree of cross-linking are thought to be more immunogenic and there have been reports of delayed onset immune-mediated nodules, although this has not been evaluated systematically in patients with connective tissue disease.^4^ In this prospective cohort study of patients with SSc, Restylane Silk (Galderma) was chosen given its low cross-linking as well as its relatively higher hydrophilicity that was hypothesized to be beneficial in fibrotic tissue.^5^ Seven patients with SSc and associated perioral changes were identified. All patients were anesthetized with compounded topical 30% lidocaine and subsequently treated with Restylane Silk between December 2020 and January 2024. Assessments were made based on photographs obtained before and immediately after injections (Fig 1). Two dermatologists (K.S., S.R.K.) independently rated baseline and posttreatment lip fullness using the Medicis Lip Fullness Scale on a 1-to-5 scale (1-very thin, 5-very full). The evaluators rated the change from baseline after treatment using the Investigator Global Aesthetic Improvement Scale, and all participants completed the Subject Global Aesthetic Improvement Scale (Fig 2). For both Investigator Global Aesthetic Improvement Scale and Subject Global Aesthetic Improvement Scale, a score of 1 represented “very much improved” and 5 represented “worse than the original condition.” The evaluators were blinded during the outcome assessment. All participants were female with a mean age of 60 and mean duration of disease of 13 years, and 4/7 patients were immunosuppressed. Participants underwent an average of 3 treatments and received an average of 1 mL of HA filler per treatment. The average time between treatments was 6 months (range, 2-16 months). The mean difference between pre- and postinjection scores on Medicis Lip Fullness Scale were 1.71 (SD 0.91, *P* < .001) for upper lip and 1.86 (SD 0.86, *P* < .001) for lower lip (Fig 3). The mean posttreatment Investigator Global Aesthetic Improvement Scale score was 2.07 (SD 0.83), and the mean posttreatment Subject Global Aesthetic Improvement Scale score was 2.29 (SD 0.95). There were no cases of delayed nodules or any reported flares of SSc. The results of this study suggest that HA fillers are safe and efficacious for improving the appearance of thin lips in patients with SSc with high subjective satisfaction reported by the patients. In the authors’ anecdotal experience, the effect of HA lip fillers appears to diminish more quickly in patients with SSc and repeat treatment every 3 to 6 months may be necessary. Further studies are needed to validate these observations. All but 1 patient also underwent treatment with hyaluronidase injection for microstomia prior to undergoing HA filler. Importantly, hyaluronidase injections were performed at least 1 month before HA filler injection to prevent breakdown of HA filler. Limitations of this study include a small sample size and short follow-up time. Further studies are needed to assess the durability of response in this patient population and the ideal HA filler to use given the heterogeneous nature of currently available products.

**Fig 1:**
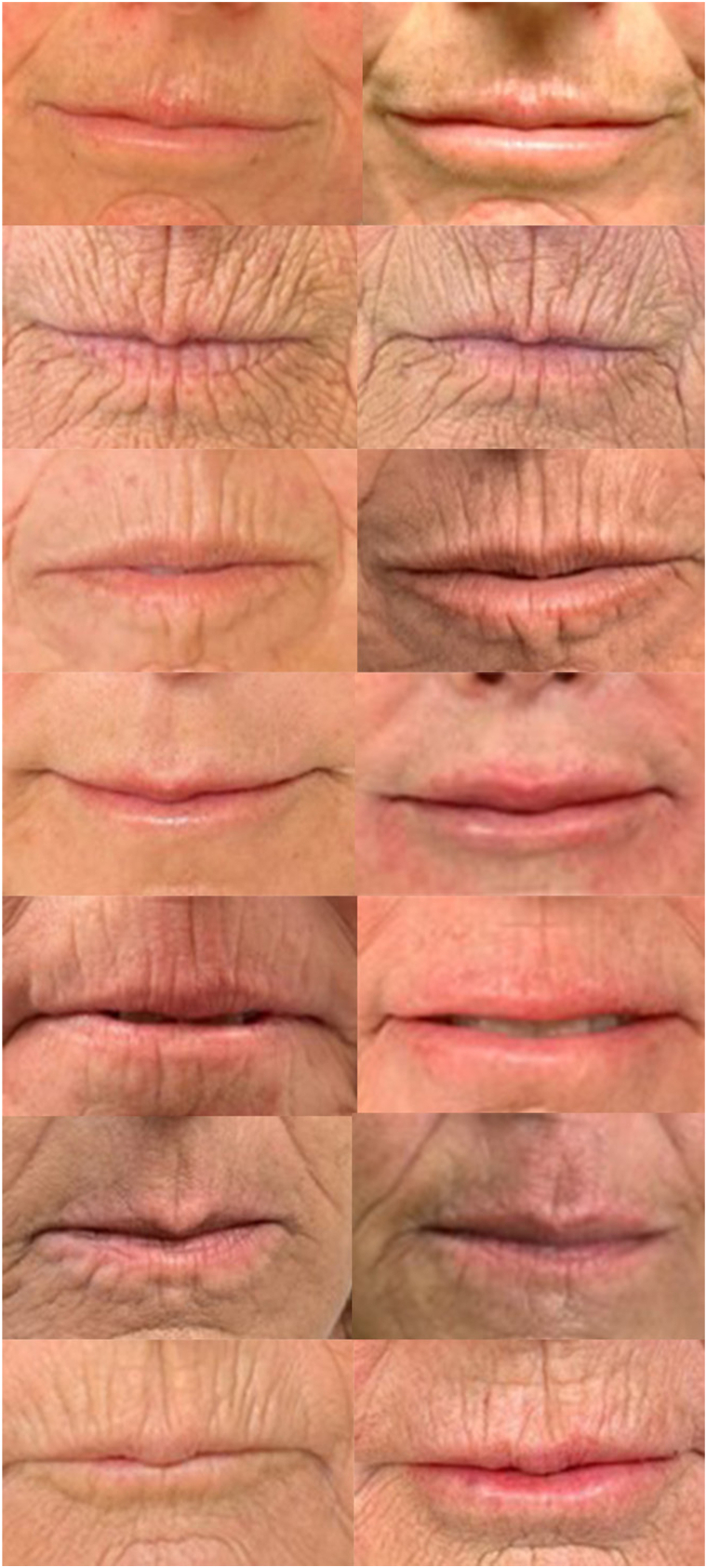
Systemic sclerosis study participants before and after treatment with hyaluronic acid filler.

**Fig 2:**
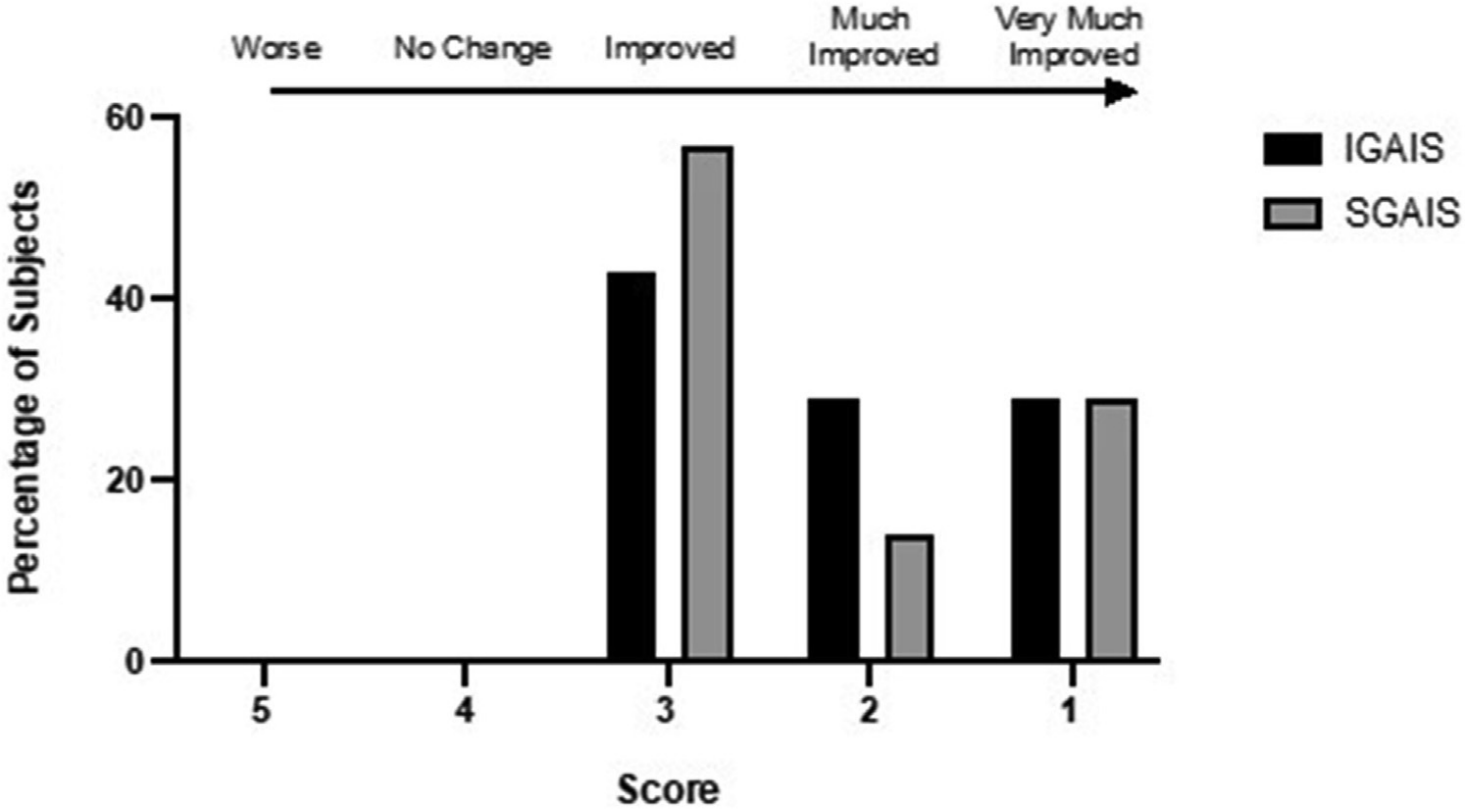
Investigator Global Aesthetic Improvement Scale (average score) and Subject Global Aesthetic Improvement Scale values following hyaluronic acid filler treatment. Score of 5: worse than the original condition, 4: no change, 3: improved, 2: much improved, and 1: very much improved. *IGAIS*, Investigator Global Aesthetic Improvement Scale; *SGAIS*, Subject Global Aesthetic Improvement Scale.

**Fig 3:**
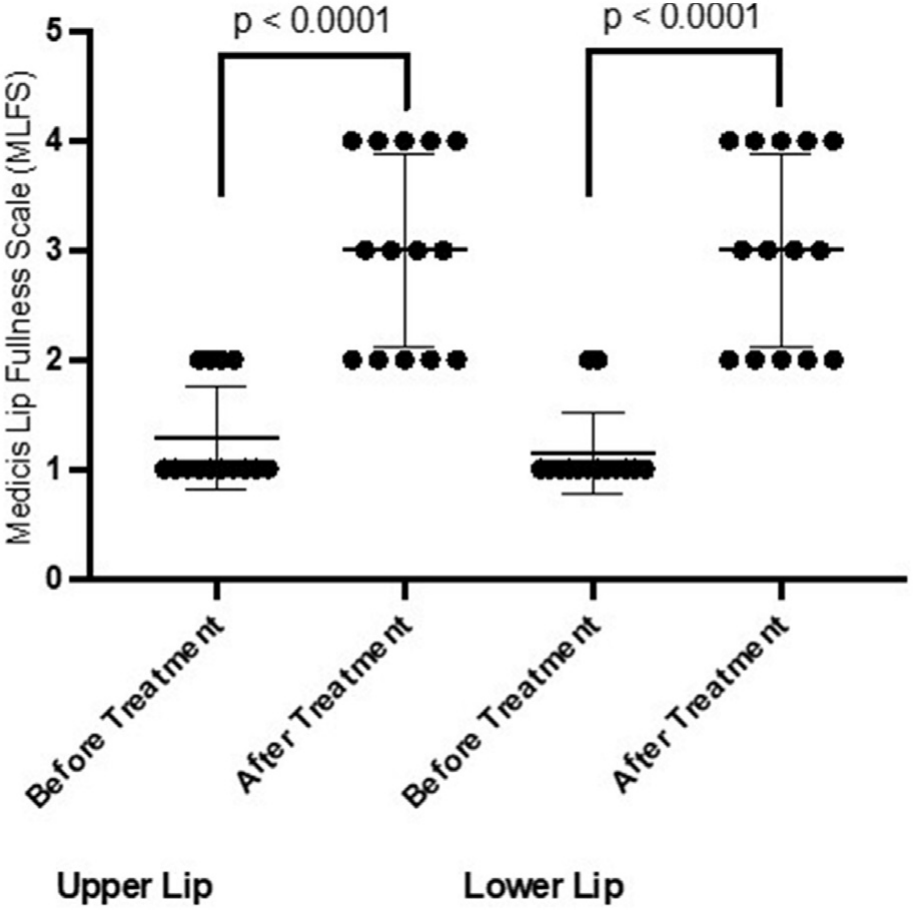
Change in Medicis Lip Fullness Scale scores following hyaluronic acid filler treatment.

## Conflicts of interest

None disclosed.
